# Conceptualization of college students’ COVID-19 related mask-wearing behaviors using the Multi-Theory Model of health behavior change

**DOI:** 10.34172/hpp.2021.24

**Published:** 2021-05-19

**Authors:** Robert E. Davis, Manoj Sharma, Kayla E. Simon, Amanda H. Wilkerson

**Affiliations:** ^1^Substance Use and Mental Health Laboratory, Department of Health, Human Performance and Recreation, University of Arkansas, 155 N Stadium Drive, HPER 308, Fayetteville, AR 72701, USA; ^2^Social and Behavioral Health, Department of Environmental & Occupational Health, School of Public Health, University of Nevada, Las Vegas, NV, USA; ^3^Department of Human Environmental Sciences, University of Alabama, 481 Russell Hall, Box 8 70311, Tuscaloosa, AL, 35487, USA

**Keywords:** COVID-19, Face mask guidelines, Policy, Social environment, University

## Abstract

**Background:** Recommendations and policies, regarding the use of face coverings, have been instituted to control transmission of coronavirus disease 2019 (COVID-19). Understanding of psychosocial factors related to the use of face coverings within the context of COVID-19 is needed. This study aimed to conceptualize mask-wearing behavior among students using the Multi-theory Model (MTM) of behavior change.

**Methods:** In October 2020, students (n = 595) enrolled in a large public southeastern US university were recruited to participate in a cross-sectional survey, using a valid and reliable instrument. Univariate, bivariate, and multivariate techniques described mask-wearing behavior and differentiated theoretical drivers of mask-wearing between individuals compliant and non-compliant with guidelines.

**Results:** Compliant individuals reported significantly higher scores (*P* <0.05) for initiation and sustenance of mask-wearing, participatory dialogue, behavioral confidence, emotional transformation, practice for change, changes in the social environment, and significantly lower scores for disadvantage. Among multivariable models, all theoretical predictors exhibited significant relationships to their respective outcomes (initiation and sustenance). Specifically, MTM constructs explained approximately 35% of variance in initiation (R^2^ = 0.346, F_(3,526)_ = 94.32, *P* <0.001) and 33% of variance in sustenance of mask wearing (R^2^ = 0.328, F_(3,529)_ = 87.71, *P* <0.001) for compliant individuals. Behavioral confidence and emotional transformation exhibited the strongest relationships to initiation (*ß* = 0.403, *P* <0.001) and sustenance (*ß* = 0.450, *P* <0.001), respectively.

**Conclusion:** Findings suggest a need to design educational programming based on the MTM to promote mask-wearing behavior among laggards who defy face mask guidelines, recommendations, and mandates.

## Introduction

The coronavirus disease 2019 (COVID-19) caused by the severe acute respiratory syndrome coronavirus 2 (SARS-CoV-2) is responsible for the current global pandemic. Currently, the United States (US) has documented more than 25 million cases and approximately 420 000 deaths due to COVID-19.^1^ SARS-CoV-2 spreads much more readily than SARS-CoV (SARS) which was responsible for a similar epidemic in 2003.^2,3^ The most recent pandemic prior to COVID-19 was due to the influenza A (H1N1) virus that occurred between 2009 and 2010.^3^ The Centers for Disease Control and Prevention (CDC) estimates between 151 700 and 575 400 deaths globally in the first year of the H1N1 pandemic.^4^ Whereas, the COVID-19 death toll surpassed 575 400 global deaths in early July 2020,^5^ just four months after officially being declared a pandemic by the World Health Organization (WHO).

Because a primary route of transmission of SARS-CoV-2 occurs through small droplets ejected when speaking, coughing, or sneezing,^4,6^ the CDC has developed individual level guidelines to prevent the spread of COVID-19.^4^ The CDC recommends that individuals wash their hands often, sanitize surfaces regularly, socially distance, and protect their mouth and nose with a face covering (i.e. mask) when around others.

The recency of the current pandemic begets an absence of literature linked to COVID-related mask-wearing behaviors. Prior to COVID-19, research dictates that mask-wearing compliance is low in areas where mask-wearing is not common practice,^7^ as is the case with the US. Preliminary findings suggest gender, age, geographic region, political affiliation, and racial differences associate with COVID-related mask-wearing.^8^ Misinformation regarding mask efficacy in the media coupled with discrepant messaging from government officials have led to confusion, instilled doubt, fostered anti-mask attitudes, and provoked defiant behaviors by some.^6,9,10^ Moreover, scant research validating cloth masks as an efficacious mechanism to prevent spread of infectious particles has propagated anti-mask attitudes, even when research suggests that, when worn properly, cloth face masks restrict the transmission of the virus from infected individuals to others.^4,6^

As intrapersonal factors affecting mask-wearing vary,^8,10^ upstream drivers of behavior such as mask mandates are important tools for increasing COVID-related mask usage.^6^ During the H1N1 epidemic, Mexico City saw an increase in compliance with face-covering guidelines following the implementation of policy mandating use.^6^ Emergent research from the US, Poland, and Australia supports the efficacy of mask mandates during the COVID-19 pandemic.^11-13^ Furthermore, interpersonal influence stemming from mask policy is found to foster compliance.^14^ Yet, as means of reinforcing mask-wearing mandates are evolving, compliance remains a highly voluntary behavior.

The novelty of COVID-related mask-wearing requires comprehensive study in order to cultivate understanding of factors related to compliance with guidelines. Theory-based interventions are shown more effective in facilitating behavior change than interventions lacking such theoretical foundation.^15^ Moreover, the Multi-Theory Model (MTM) of Health Behavior Change combines conceptual strengths from existing socio-behavioral theories and uses them to predict initiation and sustenance of health behavior change. The MTM has demonstrated efficacy in its ability to conceptualize behaviors, including, physical activity, dietary behaviors, vaccination practices, substance use, relaxation practices, intentional outdoor behaviors, and COVID-related handwashing, among others.^16-23^

Initiation of behavior change is predicted by; participatory dialogue, behavioral confidence, and changes in the physical environment. Participatory dialogue considers the advantages and disadvantages of changing behavior. Behavioral confidence focuses on an individual’s subjective confidence in their ability to institute future behavior modification. Lastly, changes in the physical environment focusses on the extent to which an individual can cultivate an environment supportive of successful behavior modification. Similarly, sustenance (i.e. maintenance) of behavior change is predicted by emotional transformation, practicing for the change, and changes in the social environment. Emotional transformation considers the individual’s ability to direct their emotions toward successful behavior modification. Practicing for change considers behavioral skills by which an individual thinks and reflects on their health behavior change. The final construct of the MTM is changes in the social environment. This construct involves the utilization of supportive social relationships in order to increase the likelihood of successful behavior maintenance.^24^

Amidst various high priority concerns, attention has been placed on college campuses during the current pandemic due to the nature of these uniquely diverse and densely populated environments. College student infection poses not only a risk of localized infection but as individuals within these environments are highly mobile (e.g. back-and-forth travel between the university and one’s home) they pose a heightened risk for widespread transmission. For instance, as college campuses resumed for fall 2020 semester activities, there was an upsurge in recorded cases of COVID-19 among young adults across the US.^25,26^ Therefore, the purpose of this study was to determine whether an evidence-based paradigm, the MTM, could explain mask-wearing behavior among college students and suggest recommendations for interventions to promote this behavior during the COVID-19 pandemic among this target group.

## Materials and Methods

### Participants and procedures

The current study utilized a cross-sectional electronic survey design. Participants were college students enrolled in a public university located within the southeastern United States (fall 2020 enrollment was approximately 28 000). Participants were recruited using convenience sampling through an advertisement in the University’s daily e-news bulletin. The advertisement ran in Thursday’s edition for three consecutive weeks in October of 2020. Inclusionary criteria required that participants be at least 18 years of age, have internet access and the ability to comprehend English, and that where able to provide informed consent. Participants exhibiting large amounts of missing data were excluded (i.e. those who provided only demographic information and failed to answer items related to study aims). The advertisement contained a brief description of the study and informed that, by participating, individuals were eligible to enter a drawing for one of five $20.00 Walmart e-gift cards. Students clicked a survey link contained in the recruitment advertisement directing them to a Qualtrics-based questionnaire. Here, participants were provided a description of study procedures including a review of their rights, anonymous nature of participation, potential risks of participation, and approved Institutional Review Board (IRB) protocol number with contact information for both the IRB and the study’s primary investigator. Moreover, participants were instructed that by clicking the ‘next’ button they acknowledged being at least 18 years of age, a current student, and providing their informed consent. The final survey item served as an invitation to enter the e-gift card drawing. A response of ‘yes’ to this item linked participants to another survey where they only provided an email address for contact purposes. This methodology allowed for the separation of previously collected data and the participant’s email address, preserving anonymity.

### Instrumentation 

A 33-item valid and reliable instrument was utilized for data collection purposes. Our behavioral focus was the wearing of face coverings or masks, as defined by the CDC, during the current COVID-19 pandemic. Thus, considerations were given to specific guidelines when constructing behavioral and theory construct measures. Six items measured previous use of face masks (yes/no), demographic characteristics including; age, gender, ethnicity, academic classification (e.g. freshman–graduate student), and work status. Specifically, previous behavior was measured using the following item, “For safe protocol during COVID-19, the CDC suggests covering the nose and mouth area using face masks (cloth/surgical/N95) be practiced when exposing oneself to public settings especially with people who do not live in your household and when there is difficulty in maintaining social distancing.^4^ Keeping in mind the above statement, did you wear cloth face coverings or masks in the past 24 hours when in public settings?”

The remaining items assessed MTM constructs. Prior to deployment, face, content, and construct validity of the instrument were established. Face and content validity were established using an expert panel including field experts in psychology, public health, and health education/promotion. Construct validity was determined by confirmatory analysis with maximum likelihood estimation. Using this method, each subscale yielded a single-factor solution, with all factor loadings over 0.32 and all Eigenvalues greater than 1.0. Cronbach α was used to establish internal consistency of the survey instrument, with acceptable reliability denoted as an α value of ≥ 0.70.^27^

The MTM is designed to explain initiation and sustenance of behavior change. Participatory dialog, behavioral confidence, and changes in the physical environment are predictive of one’s initiation of change. Participatory dialogue considers advantages and disadvantages to initiating mask-wearing behavior. Participatory dialogue ‘advantages’ were measured by five items scored on a 5-point frequency scale ranging from 1 (never) to 5 (very often). For example “If you intend to wear cloth face coverings or masks in public settings you might have less chances of getting COVID-19.” Similarly, participatory dialogue ‘disadvantages’ were measured by five items scored the same 5-point frequency scale. For example, “If you intend to wear cloth face coverings or masks in public settings you might feel inconvenienced.”Behavioral confidence refers to confidence in initiating the behavioral action of mask-wearing. This construct was measured using four items scored on a 5-point scale ranging from 1 (not at all sure) to 5 (completely sure). For example, “How sure are you that you can wear cloth face coverings or masks in public settings in the next day despite feeling discomfort?” Changes in the physical environment considers modification to the environment in order to facilitate initiation of mask-wearing. This construct was measured using three items scored on 5-point scale ranging from 1 (not at all sure) to 5 (completely sure). For example, “How sure are you that you will have access to a cloth face covering or mask every day?”

Sustenance of change is predicted by emotional transformation, practicing for change, and changes in the social environment. Emotional transformation reflects the individual’s direction of their own emotions towards the goal of mask-wearing. This construct was measured using three items scored on a 5-point scale ranging from 1 (not at all sure) to 5 (completely sure). For example, “How sure are you that you can direct your emotion/feelings toward the goal of wearing a cloth face covering or mask in public settings?” Practice for change reflects the individual’s ability to self-monitor, overcome barriers, and focus on their efforts on maintaining change. Practice for change was assessed using three items scored on a 5-point scale ranging from 1 (not at all sure) to 5 (completely sure). For example, “How sure are you that you can keep a diary/record to monitor the goal of wearing a cloth face covering or mask in public settings?” Changes in the social environment measures one’s perceived ability to utilize social resources to facilitate behavior. This construct was measured using two items scored on a 5-point scale anchored by 1 (not at all sure) and 5 (completely sure). For example, “How sure are you that you can get the help of a friend to support you with wearing a cloth face covering or mask in public settings?” Behavioral initiation and sustenance were both measured with one item scored on 5-point scale ranging from 1 (not at all sure) to 5 (completely sure). For example, “How likely are you to initiate wearing a cloth face covering or mask in public setting in the next day?” and “How likely are you to wear a cloth face covering or mask in public settings until the COVID-19 pandemic is over?” The entire instrument’s language was deemed appropriate based on the Flesch reading ease metric of 61.4 and Flesch-Kincaid Grade Level of 6.5 or less than eighth grade as is generally advocated for survey instruments.^27^ The internal consistency for the MTM scales was acceptable for all sub-scales (Cronbach’s alpha ≥ 0.70) except the practice for change items.

### Statistical analyses

Data analyses for the current study were conducted using IBM SPSS Statistics version 24.0 (IBM Corp. Armonk, NY, USA). Prior to analysis, participants exhibiting large amounts of missing data (i.e. those who provided ≤ the initial demographic items of the survey instrument) were removed (n=64). Subsequent missing data was handled using listwise deletion. For comparative purposes, the sample was split into those in compliance with mask-wearing guidelines, and those reporting non-compliance. Univariate statistics were calculated to reflect characteristics of the study sample as well as descriptors for MTM variables. Correlational analysis was used to examine bivariate relationships between MTM study variables. Additionally, Welch’s *t*tests were used to detect statistically significant differences in MTM variables between those adhering to guidelines and those who were not. Because of the small number of participants reporting non-adherence (4.5% of total sample), bootstrapping consisting of 1000 random samples with replacement was used for point estimation. Finally, multiple regression modeling was used to explain initiation and sustenance of mask-wearing among those complying with guidelines. Using G*Power version 3.1, a power analysis was conducted to determine the simple size required to conduct multiple regression modeling. Alpha was set at 0.05, power at 0.80, predictors set at 6, with effect size of 0.15 (medium). The MTM assumes 3 constructs as predictors of both initiation and sustenance models. For power analyses, 6 predictors were included to account for potential addition of covariates. Results of the power analysis dictated a minimum sample of 98, which we increased by 10% (to 108 minimum) to account for potential incomplete data. Demographic covariates were not included within regression models due to their lack of significant bivariate relationship with outcome variables. Similar modeling was not conducted among those exhibiting non-compliance with guidelines due to sample size restrictions.

## Results

Six hundred and one students were recruited for participation in the current study. Of these individuals, 6 were excluded due to large amounts of missing data. Thus, the final study sample included 595 participants (Table 1). Most participants identified as female (n = 441; 73.4%) and White (n = 428; 71.2%). Participants represented all academic classifications at the university, with the largest groups including first-year undergraduate students (n = 127; 21.1%) and graduate students (n = 189; 31.4%). Mean age among respondents was 24.86 (SD = 10.62) years, and among those reporting employment (n = 336; 55.9%), mean time worked per week was 24.30 (SD = 12.53) hours. At the time of survey administration, 94.7% (n = 559) of participants reported compliance with CDC face covering guidelines.

There were significant differences for both initiation and sustenance variables between individuals compliant with mask guidelines and those who were not (Table 2). For initiation, compliant individuals reported significantly higher mean initiation scores (*P*= 0.048), advantages-disadvantages scores (*P*= 0.041), and behavioral confidence scores (*P*= 0.005). Non-compliant individuals reported significantly higher mean disadvantages scores (*P*= 0.015). Notably, mean scores for sustenance, compliant individuals reported significantly higher mean scores for sustenance (*P*= 0.017), emotional transformation (*P*= 0.005), practice for change (*P*= 0.015), and changes in the social environment (*P*= 0.046).

Correlations between initiation and sustenance scores and all respective subscales were calculated for both compliant and non-compliant individuals (Table 3). Among compliant individuals, both initiation and sustenance scores were significantly correlated with all respective constructs (*P*< 0.001). Whereas, for individuals non-compliant with face-covering guidelines, initiation was only significantly correlated with participatory dialogue advantages-disadvantages and behavioral confidence (*P* < 0.001), and sustenance was only significantly correlated with emotional transformation (*P*< 0.001).

Multiple regression models were created for initiation and sustenance using only the individuals compliant with face covering guidelines (*n*= 559). Regression modeling for both models are presented in Table 4. For initiation, a significant regression model emerged accounting or 34.6% of variation in mask wearing (*F*_(3,526)_ = 94.32; *P* < 0.001; adjusted *R*^2^= 0.346). Participatory dialogue advantages-disadvantages (*β*= 0.117; *P =*0.010), behavioral confidence (*β*= 0.403; *P*< 0.001), and changes in the physical environment (*β*= 0.174; *P*< 0.001) were all significant predictors of initiation of mask-wearing. Behavioral confidence had the largest standardized beta coefficient (*β* = 0.403; *P* < 0.001). For every unit increase in behavioral confidence, it resulted in a 0.142 unit increase in the intention for the initiation of mask-wearing behavior among the compliant individuals. For sustenance, a significant regression model also emerged (*F*_(3,529)_ = 87.71; *P*< 0.001; adjusted *R*^2^= 0.328) and accounted for 33% of variance in maintenance of mask wearing. Emotional transformation (*β*=.450; *P*< 0.001), practice for change (*β*= 0.107; *P*= 0.017), and changes in the social environment (*β*= 0.095; *P*= 0.029) were significant predictors of sustenance of mask-wearing. Herein, emotional transformation exhibited the largest standardized beta coefficient. For a one unit increase in emotional transformation score, intentions to sustain mask wearing increased by 0.175 units.

## Discussion

This study aimed to determine whether the MTM could explain mask-wearing behavior among college students during the COVID-19 pandemic and suggest implications for practice. The study found that 94.7% of college students in our sample were adhering to the mask-wearing guidelines issued by the University at the local level and the CDC at the national level. Emergent research from China indicates that college students (n = 1599) are highly compliant (94.1%) with mask-wearing behaviors during the COVID-19 pandemic.^28^ In the Chinese study, mask-wearing was significantly associated with gender, parents’ health status, and individual attitude. Related to the generalizability of the Chinese study, the data came from researchers in Wuhan University (i.e. where the pandemic is believed to have originated). Our findings, conjoined with the Chinese study, suggest that most students seem convinced to wear masks. At the same time, it is disheartening to note that 5% of the students are still lagging and resisting wearing masks despite the growing trends in the COVID-19 pandemic. It is important to note that even after vaccination efforts are in full force, the preventive approaches in the form of wearing masks would need to continue for a very long time and the buy-in of the laggards will be essential.

Regarding MTM, as expected, the constructs in the initiation model (participatory dialogue and behavioral confidence) as well as in the sustenance model (emotional transformation, practice for change, and changes in the social environment) were higher and statistically significant for the compliant group when compared to the non-compliant group (*P* ≤ 0.05). The only construct that was not significant was “changes in the physical environment,” and that could be because the mean scores on this construct were quite high in both groups. Therefore, mask acquisition was not seen as a barrier in this sample of students. It is noteworthy that the mean participatory dialogue score was nearly three times higher in the compliant group than the non-compliant group. This finding underscores the need to convince the target population of the advantages of mask-wearing over disadvantages.

Overall, the findings provide support for the applicability of MTM in designing interventions to promote mask-wearing behavior among college students. While our study documented high adherence with mask-wearing guidelines, our sample was comprised of college students and mask-wearing behavior is low among those with less education.^9^ It is our opinion that the MTM-based approach would work among the population with lower education as well. This assertion is based on data from previous experimental studies with other behaviors such as physical activity^19^ and fruit and vegetable consumption behavior.^29^

The regression modeling of the MTM constructs among compliant individuals also supports that MTM is a potent framework to explain mask-wearing behavior among college students. In this study, 34.6% of the variance in starting mask-wearing behavior and approximately 33% variance in maintaining mask-wearing behavior was predicted by MTM constructs which is substantial for behavioral studies in health.^27^ In a related study, about handwashing behavior during the COVID-19 pandemic among college students, it was found that, similar to this study, all three constructs of MTM in the sustenance model were significant predictors and accounted for about 45% of the variance.^22^ Further, in that same study, except for changes in the physical environment, the remaining two constructs of MTM were significant in the initiation model and accounted for approximately 27% of the variance.^22^

In looking at the initiation model of MTM, the construct of behavioral confidence was significant and held the strongest relationship to initiation. Behavioral confidence is the surety in one’s ability to perform a given behavior which in this case was wearing masks. This is an important determinant and can be fostered by having multiple sources that reinforce confidence through educational programs.

Likewise, in examining the sustenance model of MTM, the construct of emotional transformation was significant and exhibited the strongest relationship to intention for maintaining mask-wearing behavior among the compliant individuals. This finding underscores that converting emotions or feelings into concrete goals is important and educational interventions promoting mask-wearing should incorporate emotional transformation concepts.

### Implications for practice

Student wellness centers, dedicated university websites started during the COVID-19 pandemic, student health services, campus recreation centers, and classrooms (remote and face-to-face) are ideal settings to promote messages on mask-wearing for college students. Messages can also be conveyed by faculty, staff, peers, student organizations, and other such channels. Most of the education in this regard can easily occur online or through m-health programs, both of which are accessible for students.

Educational programs can underscore messages regarding advantages for mask-wearing, such as decreased chances of acquiring COVID-19 and other respiratory infections, having better health, protecting family and friends, not having to miss work or school, and other possible advantages as they emerge from activities such as brainstorming or focus group discussions with student groups conducted through videoconferencing platforms such as Zoom or WebEx. At the same time, myths and potential disadvantages to mask-wearing must be dispelled in educational programs. A common disadvantage expressed by students is that of inconvenience, which can be countered by messages such as, “*A short-term inconvenience but a protection of self, family, and friends*” or similar phrases. The construct of behavioral confidence from MTM can be built by emphasizing multiple sources and having role models that promote mask-wearing behavior, such as peers and notable university leaders. Messages about overcoming discomfort for the greater good need to be promoted through peer-to-peer programs. The construct of changes in the physical environment was likely an issue in the earlier phase of the pandemic, but currently a variety of masks are easily available, often freely distributed, and affordable by most individuals.

For sustained mask-wearing behavior change, converting emotions or feelings into goals (emotional transformation), self-motivation (practice for change) and reinforcements from family, friends, influential others in life such as instructors, coaches, university officials, and medical professionals (changes in the social environment) is vital. Educational programs must incorporate these three constructs in shaping effective messages to encourage sustained mask-wearing behavior as the pandemic continues.

While most universities have policies regarding wearing masks still we saw that 5% of the students in our sample were not complying with guidelines. Thus, there is also the need for continued enforcement of policies besides educational approaches.

The study had a few shortcomings. First, we used a cross-sectional study design that has the advantage of delivering fast results but limits establishing causal linkages as temporal data are not collected. Future research studies should employ experimental designs to validate MTM to predict mask-wearing behavior. Second, we used self-reported data but for gauging attitudes that is the only tool available for researchers. Future experimental research can employ observation of behaviors after the implementation of the educational intervention. Third, we had a very small sample of individuals who were not wearing masks due to the mandated mask-wearing policy of the University. It would be interesting to follow-up on this study if the COVID-19 pandemic continues and mandates are not in place or in countries and locales where such mandates do not exist. Fourth, in our instrumentation tool, we operationalized mask-wearing behavior by a 24-hour recall on a dichotomous scale, which has the potential to influence an accurate assessment of responses. Future studies can experiment with a 7-day recall with a wider range of responses. Moreover, participant belief that mask wearing is a desirable behavior could have introduced social desirability bias. Finally, due to time constraints and urgency, we did not conduct a test-retest reliability assessment on our scale. Future researchers should establish temporal reliability before implementing an educational trial or intervention.

## Conclusion

COVID-19 continues to rage havoc globally but some college students are not adhering to the stipulated preventive guidelines that include wearing masks in public places. In our sample, 5% of college students were not complying with the guidelines despite University mandates. The fourth-generation theory, MTM, was found to be efficacious in explaining mask-wearing behavior among college students. There is a need to design educational programs based on this theory to promote mask-wearing behavior among laggard college students who still defy the mandates. It is our opinion that the MTM can also be extended in designing educational programs to other subgroups of the population who are having difficulty adhering to mask-wearing guidelines.

## Acknowledgements

We are thankful to the participants of this study for their time and our respective institutions and administrators for their kind support.

## Funding

No external funding sources were used for conducting the current research.

## Competing interests

The authors declare no conflicts of interest.

## Ethical approval

This study was reviewed and approved by the institutional review board (IRB) of the University of Arkansas (protocol # 2009281431).

## Authors’ contributions

RED and MS contributed to conceptualization and methodologic design. RED, MS, and AHW contributed to instrument development. RED and KES were responsible for data collection. RED, MS, and AHW are responsible for interpretation of data. RED, MS, KES, and AHW drafted or revised the manuscript and are responsible for important intellectual content. All named authors have approved the manuscript in its final format and agree to be accountable for all aspects of the published work.

**Table 1:** Demographic characteristics of study sample (n = 595)

	**Mean (SD)**	**No. (%)**
Age	24.86 (10.62)	
Gender		
Female		441 (73.4)
Male		143 (23.8)
Other		11 (1.8)
Race/ethnicity		
White		428 (71.2)
Non-White		166 (27.6)
Academic classification		
1^st^ year undergraduate		127 (21.1)
2^nd^ year undergraduate		73 (12.1)
3^rd^ year undergraduate		84 (14.0)
4^th^ year undergraduate		86 (14.3)
5^th^ or more year undergraduate		24 (4.0)
Graduate student		189 (31.4)
Professional degree seeking		12 (2.0)
Employment		
Employed		336 (55.9)
Non-employed		259 (43.1)
Hours worked	24.30 (12.53)	
Face covering use		
Compliant with guidelines^a^		559 (94.7)
Non-compliant with guidelines^a^		27 (4.5)

Percentage totals may not equal 100 due to missing data in the form of participant omission. ^a^Guidelines are based on recommendations for use of facial coverings when in public settings, as defined by the Centers for Disease Control and Prevention.

**Table 2:** Descriptive statistics for study variables with test of group means between face covering compliant and non-compliant individuals

	**Face covering compliant individuals (n = 569)**				**Face covering non-compliant individuals (n = 27)**				***P*** ** value**
	**Possible range**	**Observed range**	**Mean (SD)**	**Cronbach’s alpha**	**Possible range**	**Observed range**	**Mean (SD)**	**Cronbach’s alpha**	
Initiation	0–4	0–4	3.63 (0.81)	-	0–4	0–4	2.95 (1.40)	-	0.048*
Participatory dialogue: advantages	0–20	0–20	16.77 (3.72)	0.90	0–20	0–20	13.35 (6.81)	0.98	0.072
Participatory dialogue: disadvantages	0–20	0–20	6.77 (4.20)	0.81	0–20	2–20	10.00 (5.16)	0.87	0.015*
Participatory dialogue: advantages–disadvantages	-20–+20	-19–+20	10.03 (6.79)	-	-20–+20	-16–+18	3.88 (10.56)	-	0.041*
Behavioral confidence	0–16	0–16	14.47 (2.31)	0.77	0–16	3–16	11.08 (4.06)	0.80	0.005*
Changes in the physical environment	0–12	3–12	11.10 (1.62)	0.84	0–12	3–12	10.08 (2.67)	0.86	0.088
Sustenance	0–4	0–4	3.50 (0.91)	-	0–4	0–4	2.59 (1.40)	-	0.017*
Emotional transformation	0–12	0–12	10.53 (2.33)	0.89	0–12	0–12	7.70 (3.40)	0.82	0.005*
Practice for change	0–12	0–12	8.11 (2.48)	0.58	0–12	2–12	6.77 (2.60)	0.59	0.015*
Changes in the social environment	0–8	0–8	6.42 (2.15)	0.84	0–8	0–8	4.95 (3.02)	0.91	0.046*

**Table 3:** Zero-order correlation matrix of study variables

	**Construct**	**1**	**2**	**3**	**4**
**Face covering compliant individuals (n = 569)**					
1.	Initiation	-	0.430**	0.563**	0.430**
2.	Participatory dialogue advantages–disadvantages		-	0.614**	0.369**
3.	Behavioral confidence			-	0.516**
4.	Changes in the physical environment				-
1.	Sustenance	-	0.557**	0.406**	0.382**
2.	Emotional transformation		-	0.562**	0.519**
3.	Practice for change			-	0.495**
4.	Changes in the social environment				-
**Face covering non-compliant individuals (n = 27)**					
1.	Initiation	-	0.636**	0.588**	0.371
2.	Participatory dialogue advantages–disadvantages		-	0.673**	0.480*
3.	Behavioral confidence			-	0.589**
4.	Changes in the physical environment				-
1.	Sustenance	-	0.810**	0.287	0.164
2	Emotional transformation		-	0.486*	0.311
3.	Practice for change			-	0.418
4.	Changes in the social environment				-

**Table 4:** Multiple regression models for initiation and sustenance of face covering use among compliant individuals

**Initiation model**	**b**	**SE**	**B**	**p**	**LBCI**	**UBCI**
Participatory dialogue: advantages–disadvantages	0.014	0.005	0.117	0.010	0.003	0.025
Behavioral confidence	0.142	0.017	0.403	< 0.001	0.109	0.176
Changes in the physical environment	0.088	0.021	0.174	< 0.001	0.048	0.128
Model statistics: adjusted *R*^2^ = 0.346, *F*_(3,526)_ = 94.32, *P* < 0.001						
**Sustenance model**	**b**	**SE**	**B**	**p**	**LBCI**	**UBCI**
Emotional transformation	0.175	0.018	0.450	< 0.001	0.140	0.210
Practice for change	0.039	0.016	0.107	0.017	0.007	0.072
Changes in the social environment	0.040	0.018	0.095	0.029	0.004	0.076
Model Statistics: adjusted *R*^2^ = 0.328, *F*_(3,529)_ = 87.71, *P* < 0.001						

SE = standard error of the estimate; LBCI = lower bound of the 95% confidence interval; UBCI = upper bound of the 95% confidence interval.
